# Prognostic value of baseline interleukin 6 levels in liver decompensation and survival in HCC patients undergoing radioembolization

**DOI:** 10.1186/s13550-021-00791-w

**Published:** 2021-06-02

**Authors:** Osman Öcal, Juozas Kupčinskas, Egidijus Morkunas, Holger Amthauer, Kerstin Schütte, Peter Malfertheiner, Heinz Josef Klümpen, Christian Sengel, Julia Benckert, Ricarda Seidensticker, Bruno Sangro, Moritz Wildgruber, Maciej Pech, Peter Bartenstein, Jens Ricke, Max Seidensticker

**Affiliations:** 1 Department of Radiology, University Hospital, LMU Munich, Marchioninistrasse 15, 81377 Munich, Germany; 2 Institute for Digestive Research and Department of Gastroenterology, Medical Academy, Lithuanian University of Health Sciences, Kaunas, Lithuania; 3 Department of Nuclear Medicine, Charité-Universitätsmedizin Berlin, Berlin, Germany; 4 Department of Internal Medicine and Gastroenterology, Niels-Stensen-Kliniken Marienhospital, Osnabrück, Germany; 5 Department of Internal Medicine II, University Hospital, LMU Munich, Munich, Germany; 6 Department of Medical Oncology, Amsterdam University Medical Centers, University of Amsterdam, Amsterdam, the Netherlands; 7 Radiology Department, Grenoble University Hospital, La Tronche, France; 8 Department of Hepatology and Gastroenterology, Charité – Universitätsmedizin Berlin, Campus Virchow Klinikum, Berlin, Germany; 9 Liver Unit, Clínica Universidad de Navarra, Pamplona, Spain; 10 Departments of Radiology and Nuclear Medicine, University of Magdeburg, Magdeburg, Germany; 11 Department of Nuclear Medicine, University Hospital, LMU Munich, Munich, Germany

**Keywords:** Hepatocellular carcinoma, Radioembolization, Interleukin, Survival, Liver decompensation

## Abstract

**Background:**

To confirm the prognostic value of previously published baseline interleukin 6 (IL6) and IL8 cutoff values in survival and liver dysfunction in patients with advanced HCC undergoing ^90^Y radioembolization.

**Methods:**

A total of 83 patients (77 male) represented a subset of HCC patients undergoing ^90^Y radioembolization combined with sorafenib as part of the prospective multicenter phase II trial SORAMIC. IL6 and IL8 levels were determined in serum samples collected at baseline. In this post hoc analysis, we sought to confirm the prognostic value of baseline cutoff values of 6.53 pg/mL and 60.8 pg/mL for IL6 and IL8, respectively, in overall survival (OS) or liver dysfunction (grade 2 bilirubin increase) after treatment.

**Results:**

Median OS was 12.0 months. While low baseline albumin and high bilirubin values were associated with high IL6, liver cirrhosis, alcoholic liver disease, and portal vein infiltration were associated with high IL8.

In univariate analysis, high baseline IL6 and IL8 were associated with significantly shorter overall survival (7.8 vs. 19.0 months for IL6 and 8.4 vs. 16.0 months for IL8). In addition to IL values, liver cirrhosis, Child–Pugh grade, baseline albumin (< 36 g/dL), and total bilirubin (≥ 17 µmol/L), and higher mALBI grade (2b &3) values were associated with OS. At multivariate analysis, high baseline IL6 was the only independent prognostic factor for OS (HR 2.35 [1.35–4.1], p = 0.002).

Risk factors for liver dysfunction were high baseline IL6, albumin, and total bilirubin, and mALBI grade as found in univariate analysis. High baseline IL6 (HR 2.67 [1.21–5.94], p = 0.016) and total bilirubin ≥ 17 µmol/L (HR 3.73 [1.72–8.06], p < 0.001) were independently associated with liver dysfunction.

**Conclusion:**

In advanced HCC patients receiving ^90^Y radioembolization combined with sorafenib, baseline IL6 values proved to be prognostic, confirming previous findings in patients undergoing ^90^Yradioembolization. IL6 might be useful for patient selection or stratification in future trials.

**Supplementary Information:**

The online version contains supplementary material available at 10.1186/s13550-021-00791-w.

## Background

Hepatocellular carcinoma (HCC) is the most common primary liver cancer, and in up to 90% of patients, HCC develops in a cirrhotic liver [1]. The most common etiologies of liver cirrhosis are chronic hepatitis B or C infections, alcoholic liver disease, and non-alcoholic steatohepatitis [2]. Chronic viral infections, alcohol abuse, and intracellular fat accumulation interrupt the regulation of the hepatic immune system and induce liver inflammation. Chronic inflammation causes epithelial cell death, but the high regenerative capacity of the liver compensates for this damage by inducing cell proliferation. During this process, the accumulation of reactive oxygen species and DNA mutations cause hepatocarcinogenesis. Cytokine signaling, especially pro-inflammatory cytokines (such as IL6 and IL8), plays a key trigger role in inflammation [3]. Previous authors have found increased interleukin 6 levels in patients with chronic liver disease [4].

Reduced release of IL6 from Kupffer cells by inhibition of estrogen in women has been proposed as the cause of a lower incidence of HCC women as compared to men [5]. By blocking the IL6 pathway, an HCC mouse model has demonstrated reduced tumor burden in the liver, presuming as a result of decreased chronic inflammation [6]. A recent meta-analysis has demonstrated that IL6 levels are higher in HCC patients than patients with chronic liver diseases [4]. Similarly, IL-8 has been shown to mirror tumor burden in various tumors including HCC and correlate with tumor stage in HCC patients [7].

Beyond hepatocarcinogenesis, IL6 is also associated with poorer outcomes in HCC patients. The cytokines IL6 and IL8 have been shown to predict treatment response and survival after transarterial chemoembolization (TACE) in patients with primary and metastatic liver tumors [8]. A prospective exploratory study evaluating multiple cytokines has shown that a cutoff value of 6.53 pg/mL for IL6 and 60.8 pg/mL for IL8 was associated with overall survival irrespective of tumor entity after ^90^Y radioembolization (RE) in patients with HCC or metastatic disease [9].

RE delivers radionuclide embedded microspheres to the liver tumors with much higher concentrations than liver parenchyma via injection into the hepatic artery. The results of SORAfenib in combination with local MICro-therapy guided by gadolinium-EOB-DTPA-enhanced MRI (SORAMIC, EudraCT 2009–012,576-27, NCT01126645), a prospective, phase II, randomized, controlled study in HCC patients with three study arms, has been already published [10]. In the palliative arm of the study, HCC patients were randomized to sorafenib treatment either alone or combined with RE, and the addition of RE treatment failed to show benefit over sorafenib monotherapy [10]. This post hoc analysis of the palliative arm of the SORAMIC trial aimed to validate the prognostic value of previously reported baseline IL cutoff values for overall survival in patients receiving ^90^Y-radioembolization combined with sorafenib [9].

## Methods

### Study design and patient population

This study was a post hoc analysis of the palliative arm of the SORAMIC trial. Inclusion and exclusion criteria for the SORAMIC trial have been described previously [10]. In summary, patients aged 18 to 85 years with a diagnosis of HCC in the intermediate stage (BCLC B, not eligible for TACE) or the advanced stage (BCLC-C), preserved liver function (Child–Pugh scores A to B7), an Eastern Cooperative Oncology Group performance status ≤ 2 were eligible. Extrahepatic metastases were permitted if the disease was liver-dominant, and lungs were not involved. In this post hoc analysis of baseline interleukin levels, we included only patients randomized to the combination arm (RE and sorafenib) of the study. Subjects were eligible if baseline blood samples for the evaluation of IL6 and IL8 values were available.

The study protocol was approved by the institutional review boards of each participating center, and all patients gave written informed consent for study participation, including blood sampling and evaluation.

### Treatment protocol

Patients underwent RE in a lobar fashion starting from the dominant-diseased liver lobe with semi-empiric BSA method of activity prescription. In patients with bilobar disease, treatments of the contralateral lobes were performed 4–6 weeks later. Sorafenib treatment was initiated 3 days after the last RE session. The starting dose of sorafenib was 200 mg twice daily, and if tolerated, it was escalated to 400 mg twice daily after one week.

### Follow-up and laboratory analysis

There was a preplanned participation option for the translational research within the SORAMIC study, and patients were asked to participate in additional blood sampling for cytokine analysis. From patients agreeing to participate, blood samples were obtained before the initiation of the assigned treatment. Serum levels of IL-6 and IL-8 were measured with enzyme-linked immunosorbent assay (ELISA). The following ELISA kits were used in this study: Human IL-6 Quantikine ELISA Kit (R&D Sys; D6050), and Human IL-8/CXCL8 Quantikine ELISA Kit (R&D Sys; D8000C). All analytical procedures were performed according to manufacturers’ instructions. After optical density measurements at 450 nm and 570 nm (as the reference) wavelengths using Tecan Sunrise absorbance microplate reader, concentrations were calculated using a four-parameter logistic regression (4-PL) curve fitting model. By using enzyme-linked immunosorbent assays, serum levels of the IL6 and IL8 were measured. Serum IL6 and IL8 levels were defined as high or low, according to previously published cutoff values of 6.53 and 60.8 pg/mL, respectively [9].

Baseline albumin and total bilirubin values were recorded for each patient, and albumin–bilirubin (ALBI) score was calculated. Modified ALBI (mALBI) grade was used, and grade 1 was grouped together with grade 2a [11]. Within trial, patients were assessed every 2 months for a minimum of 2 years or until death, and at each visit liver function tests, including albumin and bilirubin, were repeated. The presence of any grade ≥ 2 bilirubin increases according to CTCAE (Common Terminology Criteria for Adverse Events) version 5.0 was defined as liver dysfunction. Time to liver dysfunction was recorded for each patient, and for patients with no grade ≥ 2 bilirubin increases, time to liver dysfunction was censored at the last available laboratory follow-up. The presence of RE-induced liver disease (REILD), which was defined as symptomatic ascites and jaundice (total bilirubin > 3 mg/dl) in the absence of tumor progression and biliary obstruction within the 8 weeks after RE [12], was also evaluated. Additionally, progression-free survival (PFS), based on local investigator assessment, was recorded.

### Statistical analysis

All statistical analyses were performed using R statistical and computing software, version 3.5.0 (http://www.r-project.org). Categorical variables were reported as counts and percentages, and continuous variables as means and standard deviations. Correlations were evaluated with Chi-square and Fisher’s exact tests, and t test was used to compare two groups. The Kaplan–Meier method was used for estimates of overall survival, PFS, and time-to-liver dysfunction. We employed cutoff values of 6.58 for IL6 and 60.8 for IL8 as previously reported by an exploratory analysis [9]. The accrual goal was 26 and 49 patients per IL 6 cutoff value to provide a statistical power of 80% and 90% at a significance level of 0.05. In order to eliminate the effects of disease progression on liver dysfunction, time-to-liver dysfunction analyses for IL6 and IL8 were repeated using the Kaplan–Meier method censoring patients at the time of progression who had disease progression before grade ≥ 2 bilirubin increase.

Cox regression models were used to assess the effects of cofounding factors on overall survival and liver dysfunction. Statistically significant variables in the univariate analyses were analyzed in multivariate Cox regression using two models to explore prognostic factors of overall survival and liver dysfunction. While Model 1 included albumin and total bilirubin separately, mALBI grade was used in Model 2.

## Results

### Baseline characteristics

**Fig. 1:**
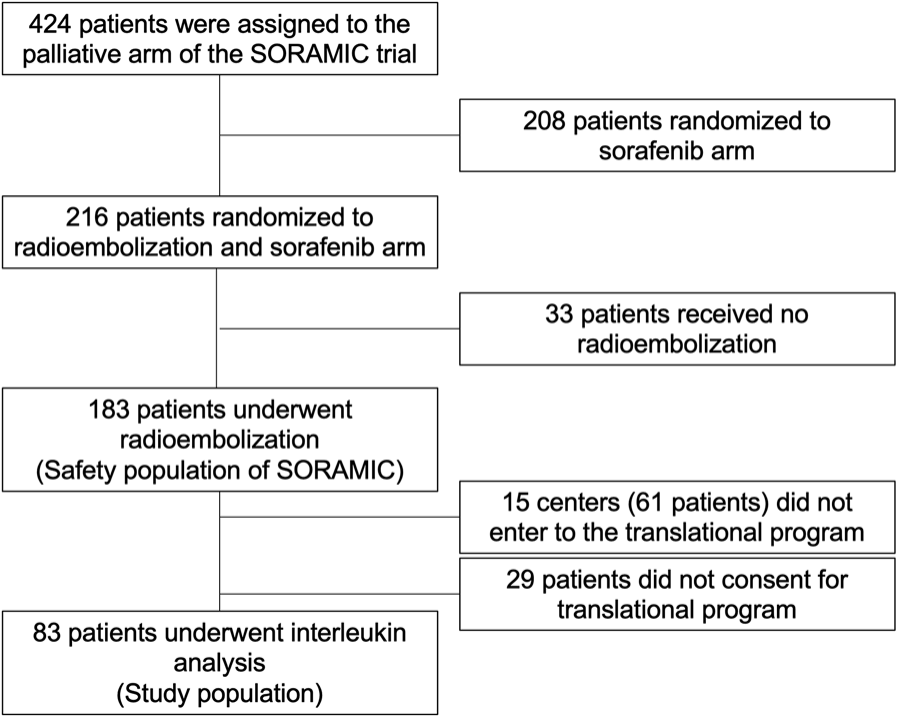
Flowchart of the study population. A total of 83 patients were analyzed in this study

Out of 424 patients included in the palliative arm of the SORAMIC trial, 216 patients were randomized to RE and sorafenib treatment. Thirty-three patients did not receive RE. Out of the remaining 183 patients, 83 (45.3%) who underwent baseline blood sampling accessible for IL assessment were the study population (Fig. 1). All of these 83 patients received sorafenib following RE. At the end of the study, 73 (87.9%) patients had died; the median OS of the post hoc study population was 12.0 (95% CI 9.7–16.0) months. Analysis according to predefined cutoffs revealed 48 (57.8%) patients with high IL6 value and 38 (45.7%) patients with high IL8.

Baseline characteristics are displayed in Table 1. There were significantly more patients with high IL6 in patients with total bilirubin ≥ 17 µmol/L (39.5% vs. 17.1%, p = 0.027), albumin < 36 g/L (35.4% vs. 8.5%, p = 0.004), and mALBI grade 2b and 3 (37.5% vs. 8.5%, p = 0.002). High IL8 was associated with liver cirrhosis (94.7% vs. 75.5%, p = 0.030), alcoholic liver disease (68.4% vs. 40.0%, p = 0.009), and portal vein invasion (52.6% vs. 24.4%, p = 0.008). Besides these, there was a trend for high IL6 in patients with cirrhosis (p = 0.065), portal vein infiltration (p = 0.061), and higher BCLC classification (p = 0.053); and a trend for high IL8 in patients with diffuse disease (≥ 10 lesions, p = 0.072), higher Child–Pugh grade (p = 0.088), and lower albumin (p = 0.054).

**Table 1:** Patient demographics and comparison of baseline characteristics of patients

	All cohort (n = 83)	IL6 low (n = 35)	IL6 high (n = 48)	p	IL8 low (n = 45)	IL8 high (n = 38)	p
Gender (male)	77 (92.7)	34 (97.1)	43 (89.5)	0.393	42 (93.3)	35 (92.1)	> 0.999
Age (≥ 65 years)	42 (50.6)	18 (51.4)	24 (50.0)	> 0.999	24 (53.3)	18 (47.3)	0.588
Race (White)	74 (89.1)	32 (91.4)	42 (87.5)	0.727	41 (91.1)	33 (86.8)	0.725
ECOG							
0	58 (69.8)	28 (80.0)	30 (62.5)	0.111	35 (77.8)	23 (60.5)	0.122
1	24 (28.9)	7 (20.0)	17 (35.4)		10 (22.2)	14 (36.8)	
Missing	1 (1.2)		1 (2.1)			1 (2.6)	
Liver cirrhosis (yes)	70 (84.3)	26 (74.2)	44 (91.6)	0.065	34 (75.5)	36 (94.7)	**0.03**
HCC etiology							
Hepatitis B	4 (4.8)	2 (5.7)	2 (4.1)	> 0.999	3 (6.6)	1 (2.6)	0.621
Hepatitis C	19 (22.8)	6 (17.1)	13 (27.0)	0.287	10 (22.2)	9 (23.6)	0.874
Alcohol	44 (53)	15 (42.8)	29 (60.4)	0.113	18 (40)	26 (68.4)	**0.009**
Previous TACE	18 (15.6)	9 (25.7)	9 (18.7)	0.447	11 (24.4)	7 (18.4)	0.507
Diffuse disease (≥ 10 lesion)	48 (49.3)	20 (57.1)	28 (58.3)	0.913	22 (48.8)	26 (68.4)	0.072
Median (mean) target lesion size, mm	68 (72.4)	66 (74.2)	70 (69.8)	0.65	56 (65.8)	73.5 (80.1)	0.13
Portal vein infiltration	31 (37.3)	9 (25.7)	22 (45.8)	0.061	11 (24.4)	20 (52.6)	**0.008**
Extrahepatic spread	21 (25.3)	6 (17.1)	15 (31.2)	0.144	11 (24.4)	10 (26.3)	0.845
Child–Pugh							
A	77 (92.7)	34 (97.1)	43 (89.5)	0.393	44 (97.7)	33 (86.8)	0.088
B	6 (7.2)	1 (2.8)	5 (10.4)		1 (2.2)	5 (13.1)	
BCLC							
B	26 (31.3)	15 (42.8)	11 (22.9)	0.053	16 (35.6)	10 (26.3)	0.365
C	57 (68.6)	20 (57.1)	37 (77.0)		29 (64.4)	28 (73.6)	
Up-to-7 criteria (outside)	71 (85.5)	29 (82.8)	42 (87.5)	0.552	37 (82.2)	34 (89.4)	0.532
Total bilirubin ≥ 17 µmol/L	25 (30.1)	6 (17.1)	19 (39.5)	**0.027**	11 (24.4)	14 (36.8)	0.22
Albumin < 36 g/L	20 (24.1)	3 (8.5)	17 (35.4)	**0.004**	7 (15.5)	13 (34.2)	0.054
AFP ≥ 400	29 (34.9)	11 (31.4)	18 (37.5)	0.519	13 (28.8)	16 (42.1)	0.235
mALBI grade (2b and 3)	21 (25.3)	3 (8.5)	18 (37.5)	**0.002**	8 (17.7)	13 (34.2)	0.129

Bold type indicates statistical significance;

IL, interleukin; ECOG, Eastern Cooperative Oncology Group; HCC, hepatocellular carcinoma; mALBI, modified albumin–bilirubin; TACE, transarterial chemoembolization; BCLC, Barcelona Clinic Liver Cancer; AFP, alfa fetoprotein

### Overall survival and treatment response

**Fig. 2:**
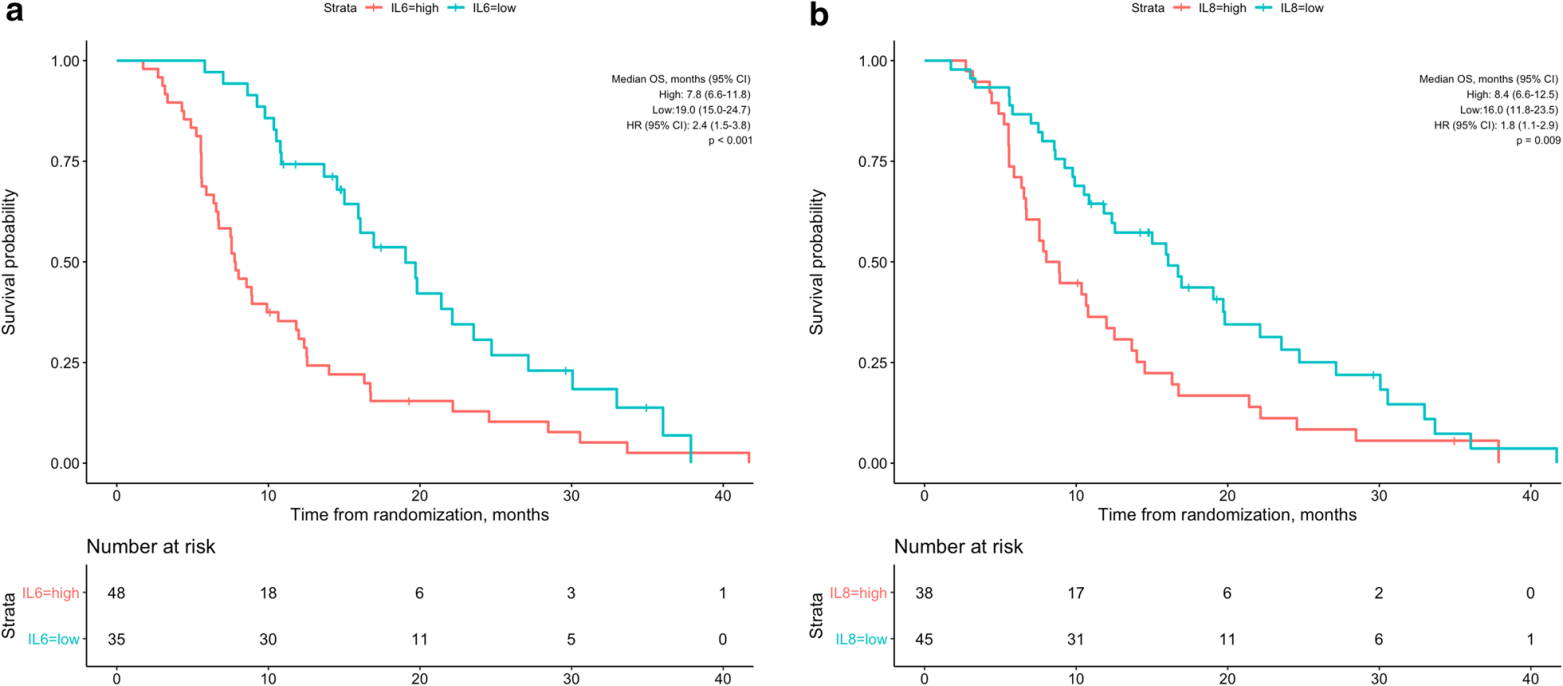
Kaplan–Meier curves showing overall survival of patients grouped by high and low baseline IL6 (**a**) and IL8 (**b**) values. P values were calculated using the log rank test

Median OS was 19.0 months (95% CI 15.0–24.7) in patients with low IL6 and 7.8 months (95% CI 6.6–11.8) in patients with high IL6 (HR 2.4 [95% CI 1.5–3.8]; p < 0.001, Fig. 2a). In addition, patients with high IL8 had significantly shorter median OS than patients with low IL8 (8.4 vs. 16.0 months, HR 1.8 [95% CI 1.1–2.9]; p = 0.009, Fig. 2b). Table 2 shows the prognostic factors associated with OS in univariate analysis. Besides high IL6 and high IL8, liver cirrhosis (p = 0.032), Child–Pugh class B (p = 0.014), albumin < 36 g/L (p = 0.024), total bilirubin ≥ 17 µmol/L (p = 0.009), and higher (2b and 3) mALBI grade (p = 0.007) were associated with worse outcome. Multivariate Cox regression analysis using Model 1 (excluding ALBI grade in order to avoid interactions) revealed high IL6 (HR 2.35, [95% CI 1.35–4.1] p = 0.002) as the only independent prognostic factor for shorter overall survival (Table 2). There was a tendency for shorter survival in patients with cirrhosis (HR 2.33, [95% CI 0.94–5.81] p = 0.069), Child–Pugh B (HR 2.91, [95% CI 0.98–8.64] p = 0.055), high total bilirubin (HR 0.58, [95% CI 0.32–1.05] p = 0.073). Similarly, high IL6 (HR 2.2, [95% CI 1.28–3.8] p = 0.005) was the only significant variable in Model 2 (including mALBI grade), and although the difference was not statistically significant, cirrhosis (HR 2.45, [95% CI 0.99–6.1] p = 0.053) and Child–Pugh B (HR 2.69, [95% CI 0.98–7.34] p = 0.054) were associated with shorter overall survival. Additionally, in separate models using each of IL6, IL8, albumin, bilirubin, and ALBI score as continuous variables, IL6 maintained the significant association with overall survival (Additional file 1: Figures S1a–e).

**Table 2:** Univariate and multivariate analysis of factors associated with overall survival

Parameter	Univariate analysis		Multivariate analysis			
			Model 1^a^		Model 2^b^	
	HR (95% CI)	p value	HR (95% CI)	p value	HR (95% CI)	p value
IL6 (> 6.53 pg/mL)	2.4 (1.5–3.88)	** < 0.001**	2.35 (1.35–4.1)	**0.002**	**2.2 (1.28–3.8)**	**0.005**
IL8 (> 60.8 pg/mL)	1.8 (1.1–2.9)	**0.011**	1.32 (0.77–2.3)	0.305	1.2(0.71–2.0)	0.481
Sex (Male vs. Female)	0.59 (0.23–1.5)	0.26				
Age (≥ 65 vs. < 65 years)	0.8 (0.5–1.3)	0.36				
ECOG (1 vs. 0)	0.96 (0.57–1.6)	0.87				
Cirrhosis (Yes vs. No)	2.4 (1.1–5.2)	**0.032**	2.33 (0.94–5.81)	0.069	2.4 (0.99–6.1)	0.053
Hepatitis B Etiology (Yes vs. No)	1.5 (0.53–4)	0.47				
Hepatitis C Etiology (Yes vs. No)	1.3 (0.73–2.2)	0.39				
Alcohol Etiology (Yes vs. No)	1.2 (0.78–2)	0.36				
Previous TACE (Yes vs. No)	0.87 (0.5–1.5)	0.63				
PVI (Yes vs. No)	1.3 (0.81–2.1)	0.28				
Child–Pugh (B vs. A)	3.3 (1.3–8.5)	**0.014**	2.91 (0.98–8.64)	0.055	2.7 (0.98–7.3)	0.054
BCLC (C vs. B)	1.1 (0.53–2.3)	0.78				
Albumin (< 36 g/L)	1.9 (1.1–3.3)	**0.024**	0.68 (0.33–1.43)	0.31	–	–
Total bilirubin (≥ 17 µmol/L)	2 (1.2–3.2)	**0.009**	1.71 (0.95–3.09)	0.073	–	–
AFP (≥ 400 vs < 400 ng/mL)	0.86 (0.53–1.4)	0.53				
Diffuse disease (≥ 10 lesions)	0.77 (0.48–1.2)	0.28				
Extrahepatic disease	0.96 (0.57–1.6)	0.89				
mALBI grade (2b and 3 vs. 1 and 2a)	2.1 (1.2–3.6)	**0.007**	–	–	1.13 (0.62–2.07)	0.694

Bold type indicates statistical significance;

IL, interleukin; ECOG, Eastern Cooperative Oncology Group; TACE, transarterial chemoembolization; PVI, Portal vein invasion; BCLC, Barcelona Clinic Liver Cancer; AFP, alfa fetoprotein; mALBI, modified albumin–bilirubin

^a^Model 1 was identified using Cox regression with albumin and total bilirubin, excluding mALBI grade

^b^Model 2 was identified using Cox regression with mALBI grade as a composite factor, excluding albumin and total bilirubin

PFS information was missing in one patient. Patients with high IL-6 had significantly shorter progression-free survival than patients with low IL6 (Additional file 1: Figure S2a; 5.5 vs. 17.9, p < 0.001). Similarly, patients with high IL8 had shorter progression-free survival (Additional file 2: Figure S2b; 7.8 vs. 15, p = 0.017) than patients with low IL6.

### Liver dysfunction

**Fig. 3:**
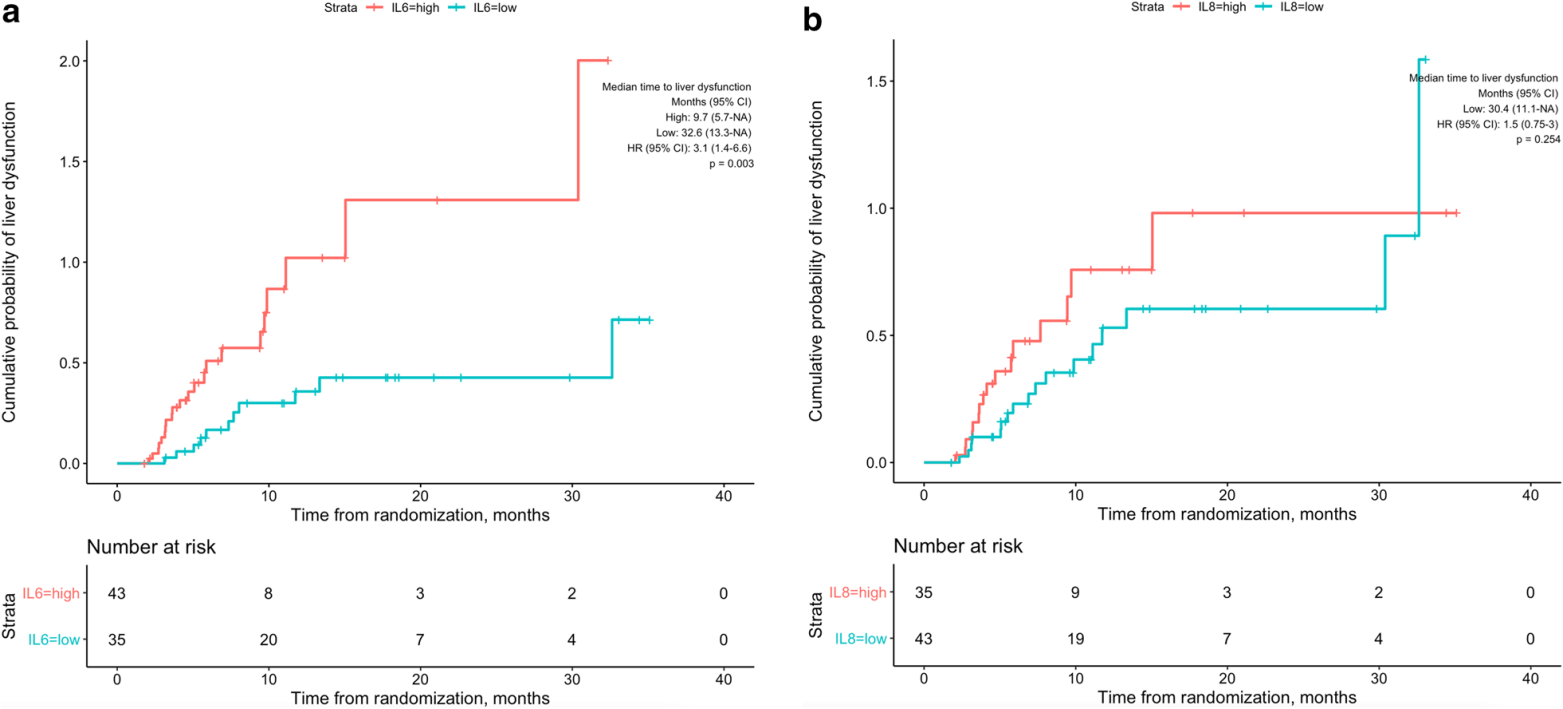
Kaplan–Meier curves showing time-to-liver dysfunction of patients grouped by high and low baseline IL6 (**a**) and IL8 (**b**) values. P values were calculated using the log rank test

Follow-up bilirubin values were available in 78 patients. There was no case of REILD. Liver dysfunction (grade ≥ 2 bilirubin increase) was seen in 33 (42.3%) patients. Patients with high IL6 had significantly shorter time to liver dysfunction than patients with low IL6 (Fig. 3a; 9.7 vs. 32.6 months, HR 3.1 [95% CI 1.4–6.6]; p = 0.003). Although there was a tendency for a shorter time-to-liver dysfunction in patients with high IL8 (9.7 vs. 30.4 months), the result was not significant (Fig. 3b; p = 0.25). In addition to IL6, low albumin (HR 3.0, [95% CI 1.4–6.3]; p = 0.003), high total bilirubin values (HR 4.4, [95% CI 2.2–9.0]; p < 0.001), and higher mALBI grade (HR 3.9, [95% CI 1.9–8.1]; p < 0.001) were associated with shorter time-to-liver dysfunction. In Model 1, multivariate analysis revealed that high IL6 (HR 2.67, [95% CI 1.21–5.94], p = 0.016) and high total bilirubin values (HR 3.73, [95% CI 1.72–8.06], p < 0.001) were independent prognostic factors of liver dysfunction (Table 3). In Model 2, both of high IL6 (HR 2.5, [95% CI 1.1–5.4], p = 0.024) and higher (2b and 3) mALBI grade (HR 3.1, [95% CI 1.5–6.5], p = 0.003) were associated with liver dysfunction.

**Table 3:** Univariate and multivariate analysis of factors associated with liver dysfunction

Parameter	Univariate analysis		Multivariate analysis			
			Model 1^a^		Model 2^b^	
	HR (95% CI)	p value	HR (95% CI)	p value	HR(95% CI)	p value
IL6 (> 6.53 pg/mL)	3.1 (1.4–6.6)	**0.003**	2.67 (1.21–5.94)	**0.016**	**2.5 (1.1–5.4)**	**0.024**
IL8 (> 60.8 pg/mL)	1.5 (0.75–3)	0.25				
Sex (Male vs. Female)	0.66 (0.2–2.2)	0.49				
Age (≥ 65 vs. < 65 years)	0.99 (0.5–2)	0.97				
ECOG (1 vs. 0)	0.84 (0.38–1.9)	0.68				
Cirrhosis (Yes vs. No)	2 (0.62–6.6)	0.25				
Hepatitis B Etiology (Yes vs. No)	1.3 (0.17–9.3)	0.82				
Hepatitis C Etiology (Yes vs. No)	1.9 (0.88–3.9)	0.1				
Alcohol Etiology (Yes vs. No)	1.1 (0.54–2.2)	0.82				
Previous TACE (Yes vs. No)	1.6 (0.73–3.3)	0.25				
PVI (Yes vs. No)	1.4 (0.68–2.9)	0.37				
Child–Pugh (B vs. A)	3.3 (0.98–11)	0.053				
BCLC (C vs. B)	1.1 (0.53–2.3)	0.78				
Albumin (< 36 g/L)	3.0 (1.4–6.3)	**0.003**	1.41 (0.61–3.23)	0.421	–	–
Total bilirubin (≥ 17 µmol/L)	4.4 (2.2–9)	** < 0.001**	3.73 (1.72–8.06)	** < 0.001**	**–**	**–**
AFP (≥ 400 vs < 400 ng/mL)	1.6 (0.73–3.4)	0.25				
Diffuse disease (≥ 10 lesions)	0.68 (0.34–1.3)	0.27				
Extrahepatic disease	1.1 (0.53–2.5)	0.74				
mALBI grade (2b and 3 vs. 1 and 2a)	3.9 (1.9–8.1)	< 0.001	**–**	**–**	**3.1 (1.5–6.5)**	**0.003**

Bold type indicates statistical significance;

IL, interleukin; ECOG, Eastern Cooperative Oncology Group; TACE, transarterial chemoembolization; PVI, Portal vein invasion; BCLC, Barcelona Clinic Liver Cancer; AFP, alfa fetoprotein; mALBI, modified albumin–bilirubin

^a^Model 1 was identified using Cox regression with albumin and total bilirubin, excluding mALBI grade

^b^Model 2 was identified using Cox regression with mALBI grade as a composite factor, excluding albumin and total bilirubin

Out of 33 patients who had liver dysfunction, only four patients were diagnosed with disease progression at the time of liver dysfunction. Time-to-liver dysfunction analysis was repeated, censoring these four patients at the time of disease progression in order to eliminate effects of tumor progression in deterioration of liver function. Similar to the first analysis, high IL6 was significantly associated with shorter median time-to-liver dysfunction (Additional file 3: Figure S3a; 9.7 [5.87-NA] vs. NA [32.6-NA] months, p = 0.01). Also, although the patients with high IL8 had shorter median time-to-liver dysfunction (Additional file 3: Figure S3b; 9.7 [5.87-NA] vs. 32.6 [11.74-NA] months, p = 0.22), the difference was not significant.

## Discussion

Our results have shown that baseline IL6 values are independent prognostic factor for overall survival and liver dysfunction in advanced HCC patients who received RE combined with sorafenib. IL8 was associated with overall survival, although the statistical significance was lost in multivariate analysis with other prognostic factors. Also, IL6 and IL8 were significantly associated with markers of advanced disease and worse liver functions.

IL6 plays a crucial role in the acute inflammatory reactions and stimulates the production of acute-phase reactants in the liver. Long-term increased IL6 levels lead to increased proliferation, resistance to apoptosis, chemoresistance, and metastatic potential in HCC [14]. IL8 is a macrophage-derived angiogenesis mediator and proinflammatory chemotactic factor for neutrophils that enhances tumor cell growth and promotes angiogenesis [7, 15]. Similar to previous reports, high IL6 and IL8 values were associated with advanced tumor stage and impaired liver functions in our study [7, 16, 17]. In our study, high IL6 values were associated with high total bilirubin, low albumin, and high mALBI grade; and high IL8 values were associated with liver cirrhosis, alcoholic liver disease, and portal vein invasion.

Our study confirms previous results of an exploratory study investigating the correlation between multiple cytokines and treatment outcomes in patients receiving RE, which showed with cutoff values of 6.53 and 60.8 pg/mL, IL6 and IL8 values could predict survival [9]. Up to date, no other study to validate these cut-off values have been reported.

RE has been proposed as an alternative treatment option for HCC patients with liver dominant disease who are not candidates for potentially curative treatments or cannot tolerate systemic therapies [2]. Although three randomized trials have failed to show superiority or additional benefit of RE over sorafenib [10, 18, 19], a recent meta-analysis of these trials has suggested non-inferiority to sorafenib, and also higher tolerability of RE [20].

High baseline IL6 values predict recurrence after resection in early-stage HCC patients [21]. However, there are contradictory reports on prognostic value of IL after locoregional therapies. In a report of 22 patients (seven had HCC) received RE, patients with more than six months of survival had significantly lower baseline IL8 values, but there was no significant difference in IL6 values [22]. Another study evaluated patients who underwent TACE, and while post-intervention (day 1) IL6 values were significantly associated with survival, baseline values were not [23]. The largest reported cohort (110 patients) evaluating the association between IL6 values and survival in HCC patients after TACE showed baseline IL6 values > 10 pg/mL is significantly associated with poor overall survival [16]. A study explored IL6 in an Asian cohort of patients with HCC (55 and 73 patients in exploration and validation cohorts) receiving sorafenib showed a cutoff value of 4.28 pg/mL is correlated with survival [24]. These differences in the outcome might be a result of low sample size or retrospective nature of the studies.

Our study confirmed the association between survival and previously reported cutoff values for IL6 (6.53 pg/mL) and IL8 (60.8 pg/mL) in patients who underwent RE followed by sorafenib. The same cutoff value for IL6 was also correlated with liver dysfunction. Although most of the evaluated patients had liver functions precluding inclusion to HCC trials, a recent study has shown a cutoff value of 7.0 pg/mL for IL6 is correlated with clinical decompensation in patients with advanced chronic liver disease [25]. The same study also showed IL6 values are independent predictors of a need for liver transplantation or death. Another study that retrospectively evaluated patients with end-stage liver disease showed IL6 values have a similar predictive value of 90-day and 1-year mortality with MELD score [26]. There were no cases with REILD in our cohort, and liver dysfunction was defined as grade 2 bilirubin increase to detect more subtle changes in liver function seen in patients after RE [12]. IL6 was significantly associated with liver dysfunction. While in patients with high IL6, median time-to-liver dysfunction was 9.7 months; in patients with low IL6, it was 32.6 months. Furthermore, IL6 was independently associated with liver dysfunction in multivariate analysis. Only four of 33 patients with liver dysfunction were already diagnosed with tumor progression at that time. To eliminate the role of tumor progression in liver dysfunction, time-to-liver dysfunction analysis was repeated, censoring these four patients at the time of tumor progression. It showed that the association between IL6 and deterioration in liver function is independent of HCC progression. A previous study has shown that RE induces a sustained increase in circulating IL6 and IL8 and activates inflammation and coagulation cascade, which has been suggested as pathogenic steps of REILD [27], and higher baseline values might be amplified after RE. Considering this study with our findings together suggests that high baseline IL6 values might be related to higher toxicity after RE. Besides, high baseline IL6 and IL8 were associated with disease progression. PFS was significantly shorter in patients with high IL6, as well as in patients with high IL8. In summary, our results show that IL6 is associated with deterioration in liver function independently from tumor progression, as well as progression-free survival. The prognosis of patients with HCC is heavily linked to liver function and tumor burden, and our findings show that IL6 could serve as a marker of the synthesis of both.

Since IL6 plays an essential role in immunity, cell proliferation, and differentiation, several therapeutics have been evaluated to suppress IL6 production or signaling pathways. Initial phase I and II studies have been promising results in Castleman’s disease or renal cell carcinoma [28, 29]. Combining anti-IL6 agents with current therapies or suppressing these pathways before initiation of treatment in HCC patients with high baseline IL6 should be evaluated, and currently tocilizumab (anti-IL6) is under investigation in patients with HCC (MORPHEUS-liver trial, EudraCT 2020-001743-10).

Our results confirmed baseline IL6 as an independent prognosticator of survival and liver dysfunction in patients with advanced HCC. Biomarkers play a key role in the decision-making process in many tumor types, but the evaluation of many biomarkers have failed to predict treatment benefit in HCC patients [30]. Baseline IL6 values might be used to predict treatment benefit and can be integrated into prognostic calculators [31, 32], and patients can be allocated to more aggressive treatments or not according to high or low IL6 values. Besides this, baseline measurements of IL6 should be used to stratify patients between treatment arms in future phase 3 trials for new drugs to improve patient selection for the therapy and avoid confounders. An additional advantage of IL6 analysis is the reasonable price which is around 10–15 EUR and hence significantly less than other recently proposed markers [33, 34].

This study has some limitations. IL sampling was not mandatory in the SORAMIC trial, and samples were not available in all recruited patients. Also, the initial study [9], which reported the cutoff values, included patients with different tumor types, and only patients with good liver functions (Child–Pugh A). Moreover, the study population had a narrow margin of tumor burden, a limitation for the prognostic value of IL8, which has been shown to correlate with tumor burden. Additionally, although HCC patients also received additional therapies in that study, all patients evaluated in this post hoc analysis received ^90^Y radioembolization combined with sorafenib. Also, further research is needed to clarify if IL6 and IL8 could serve as predictors of therapy benefit, however, this is beyond the scope of this analysis. Despite all these limitations, our study provides validation to previously published cutoff value for IL6 in a multinational prospective cohort.

## Conclusions

Baseline IL6 value is an independent prognostic factor for overall survival in HCC patients treated with ^90^Y radioembolization and sorafenib. IL6 could therefore be used as a stratification factor in future clinical trials of radioembolization. Furthermore, IL6 could be studied as part of prognostic tools to improve patient selection.

## Supplementary Information

**Additional file 1.** Supplementary figure 1. Kaplan-Meier curves showing progression-free survival of patients grouped by high and low baseline IL6 (a) and IL8 (b) values. P values were calculated using the log rank test.

**Additional file 2.** Supplementary figure 2. Results of seperate multivariable analysis models using following parameters as continous variables: (a) IL6, (b) IL8, (c) Albumin, (d) Bilirubin, (e) ALBI score.

**Additional file 3.** Supplementary figure 3. Kaplan-Meier curves showing cumulative probability of liver dysfunction without disease progression according to baseline IL6 (a) and IL8 (b) values. P values were calculated using the log rank test.

## Data Availability

Data are available from the corresponding author on reasonable request.

## Abbreviations

BCLC: Barcelona Clinic Liver Cancer
CTCAE: Common Terminology Criteria for Adverse Events
ELISA: Enzyme-linked immunosorbent assay
HCC: Hepatocellular carcinoma
IL: Interleukin
OS: Overall survival
PFS: Progression-free survival
RE: ^90^Y radioembolization
TACE: Transarterial chemoembolization
