# Supplementary material for: Prognostic value of baseline interleukin 6 levels in liver decompensation and survival in HCC patients undergoing radioembolization
Source: EJNMMI Res. 2021 Jun 2;11:51. doi: 10.1186/s13550-021-00791-w (PMC8172845; doi:10.1186/s13550-021-00791-w)
Supplement: Supplementary file 1 — Additional file 1. Supplementary figure 1. Kaplan-Meier curves showing progression-free survival of patients grouped by high and low baseline IL6 (a) and IL8 (b) values. P values were calculated using the log rank test. [file 13550_2021_791_MOESM1_ESM.pdf]

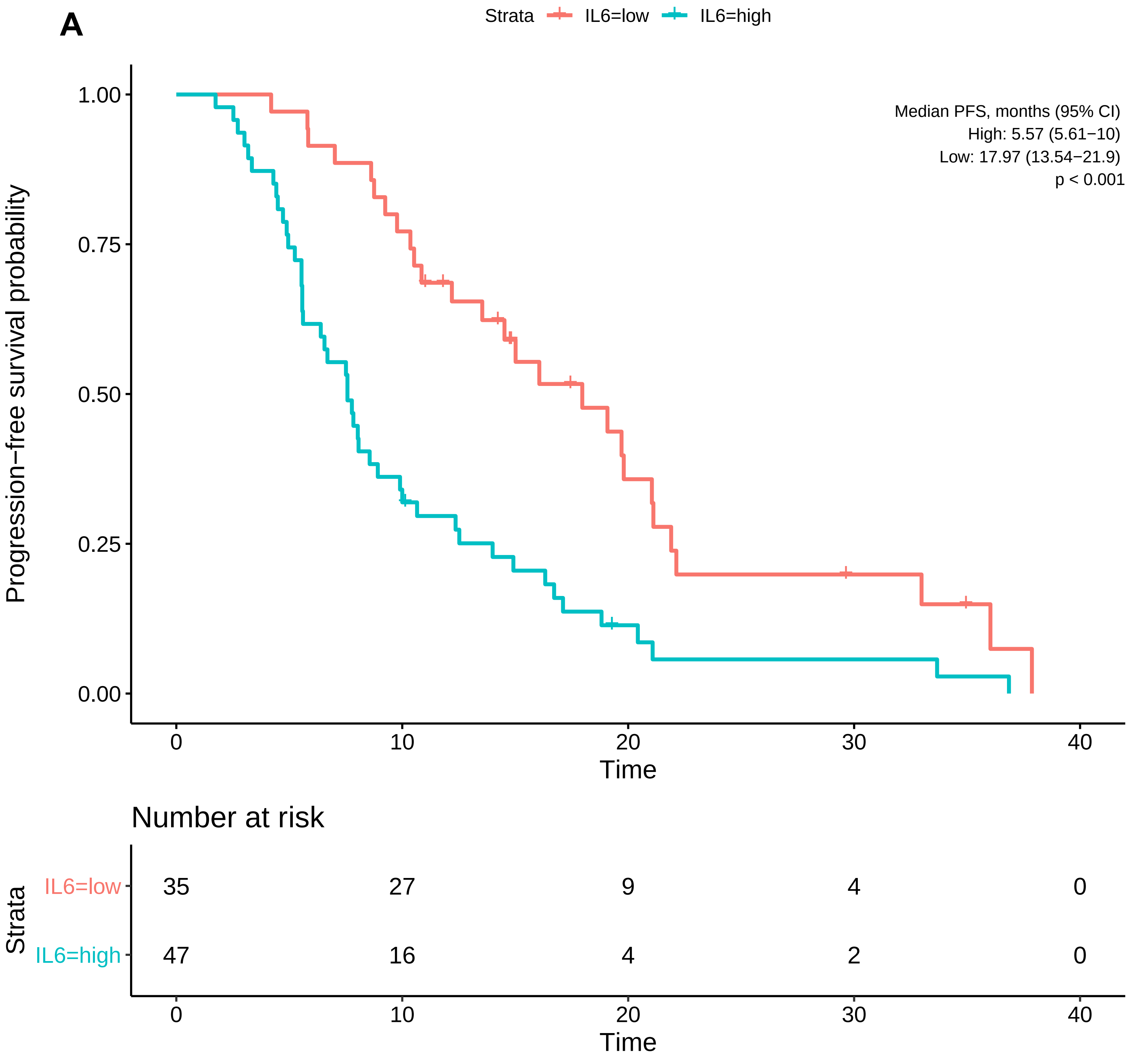

A
Strata
IL6=low
IL6=high
Median PFS, months (95% CI)
High: 5.57 (5.61−10)
Low: 17.97 (13.54−21.9)
p < 0.001
Progression−free survival probability
1.00
0.75
0.50
0.25
0.00
0
10
20
30
40
Time
Number at risk
Strata
IL6=low 35 27 9 4 0
IL6=high 47 16 4 2 0
0
10
20
30
40
Time

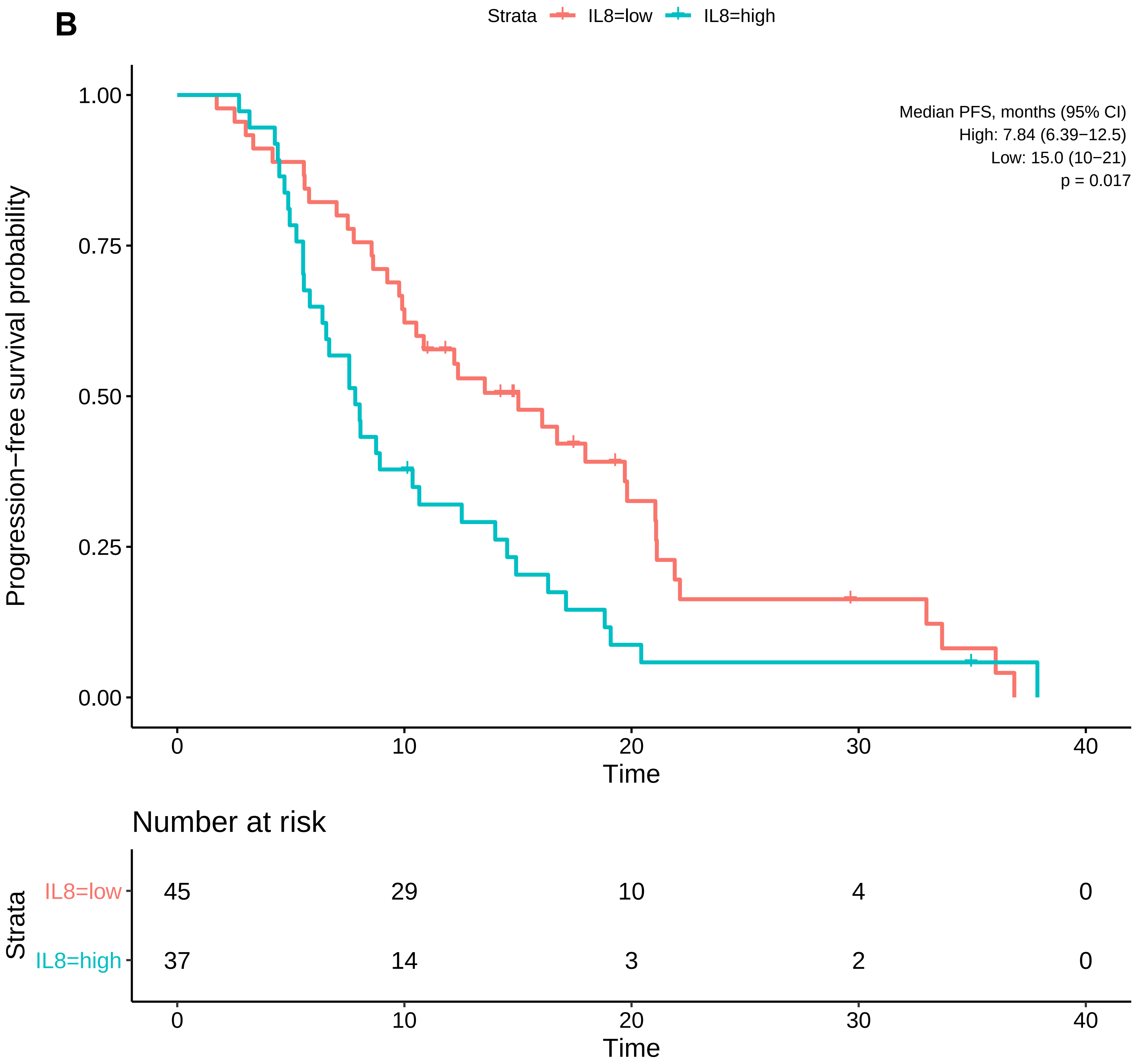

B
Strata
IL8=low
IL8=high
Progression−free survival probability
1.00
0.75
0.50
0.25
0.00
Median PFS, months (95% CI)
High: 7.84 (6.39−12.5)
Low: 15.0 (10−21)
p = 0.017
0
10
20
30
40
Time
Number at risk
Strata
IL8=low
IL8=high
45
29
10
4
0
37
14
3
2
0
0
10
20
30
40
Time
