# Supplementary material for: Prognostic value of baseline interleukin 6 levels in liver decompensation and survival in HCC patients undergoing radioembolization
Source: EJNMMI Res. 2021 Jun 2;11:51. doi: 10.1186/s13550-021-00791-w (PMC8172845; doi:10.1186/s13550-021-00791-w)

Supplementary figure 2a.
Multivariable analysis using IL6 as continuous variable

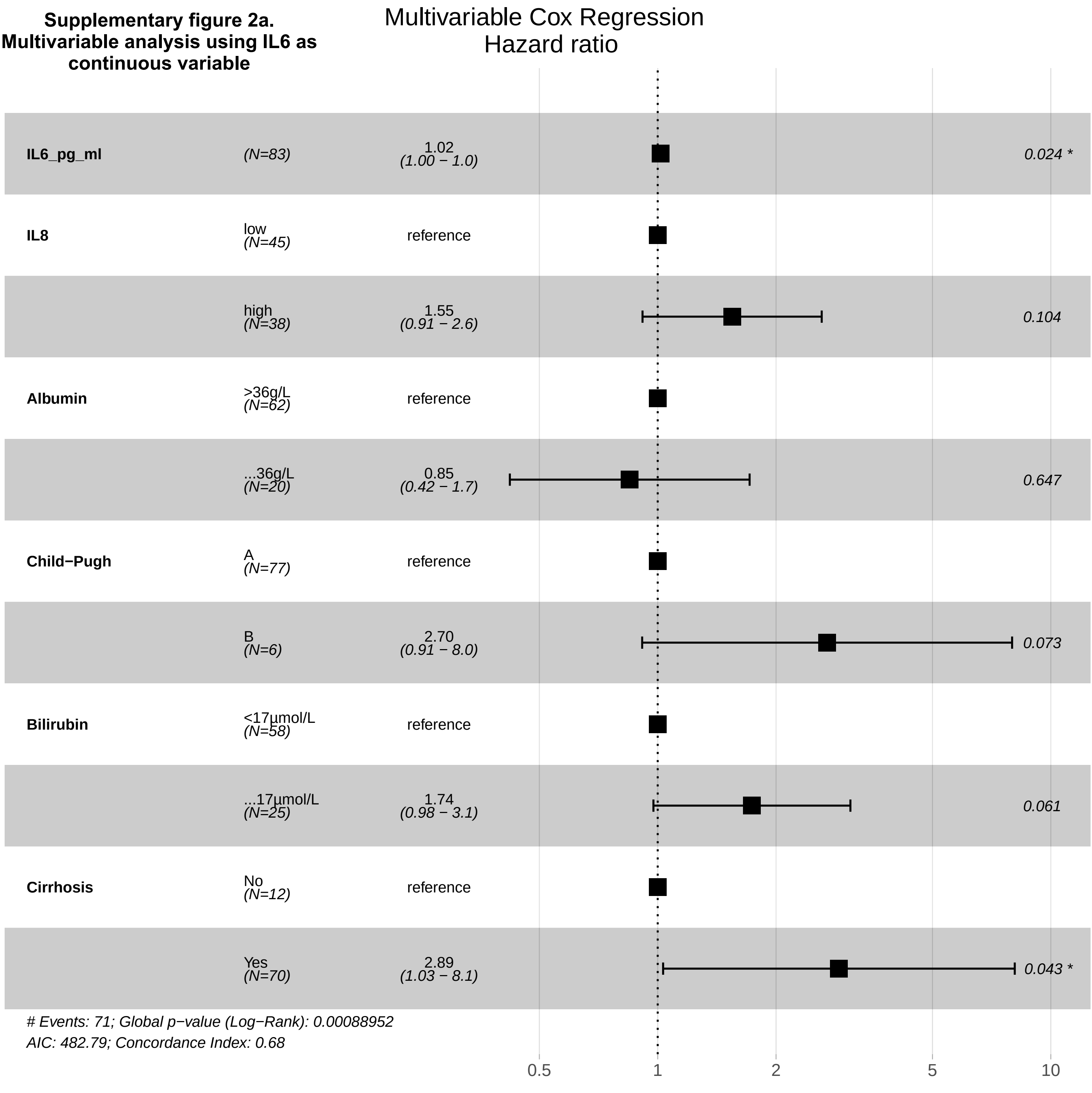

**Supplementary figure 2b.
Multivariable analysis using IL8 as continuous variable**

Multivariable Cox Regression
Hazard ratio

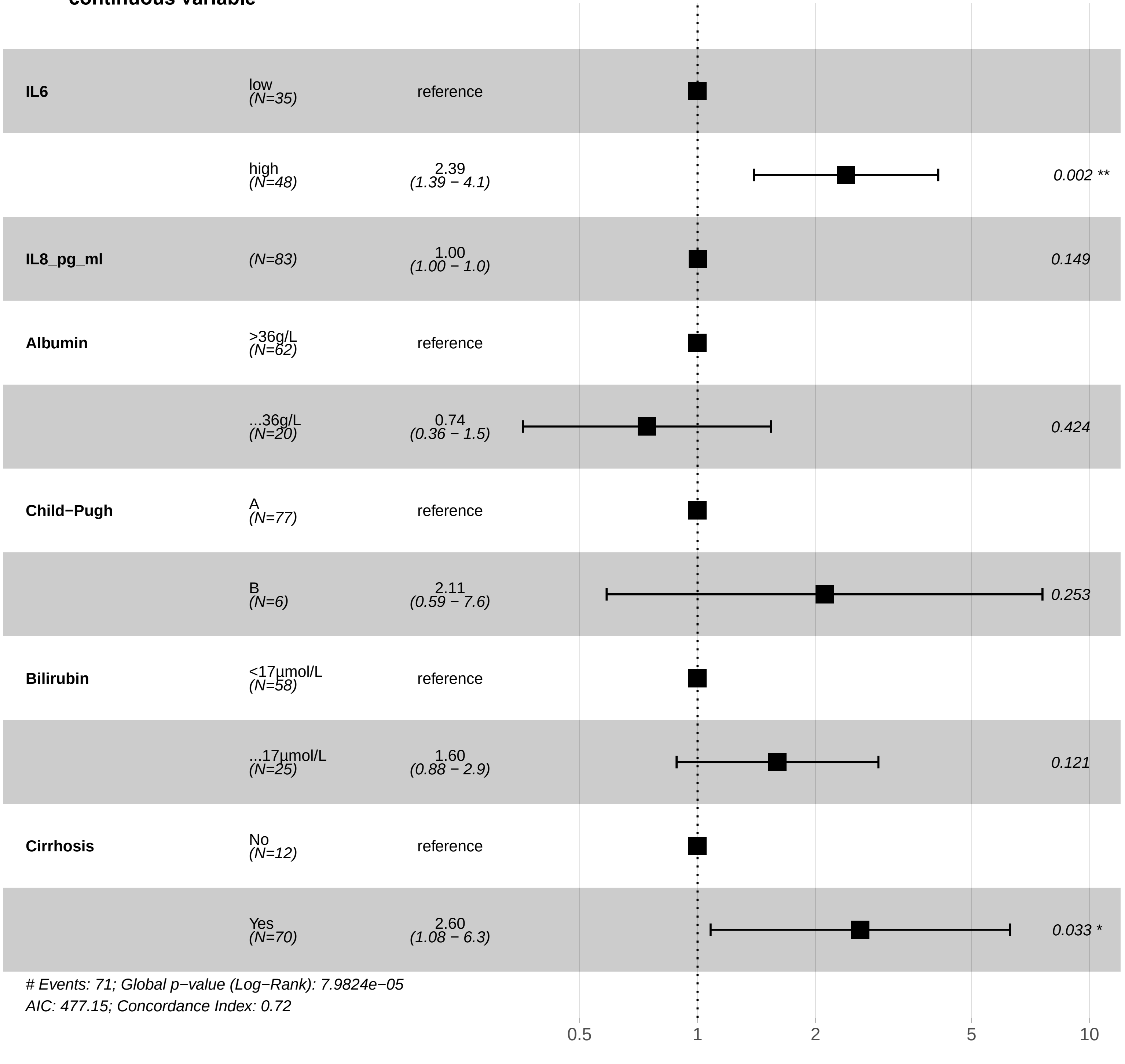

Supplementary figure 2c.
Multivariable analysis using albumin as continuous variable

Multivariable Cox Regression
Hazard ratio

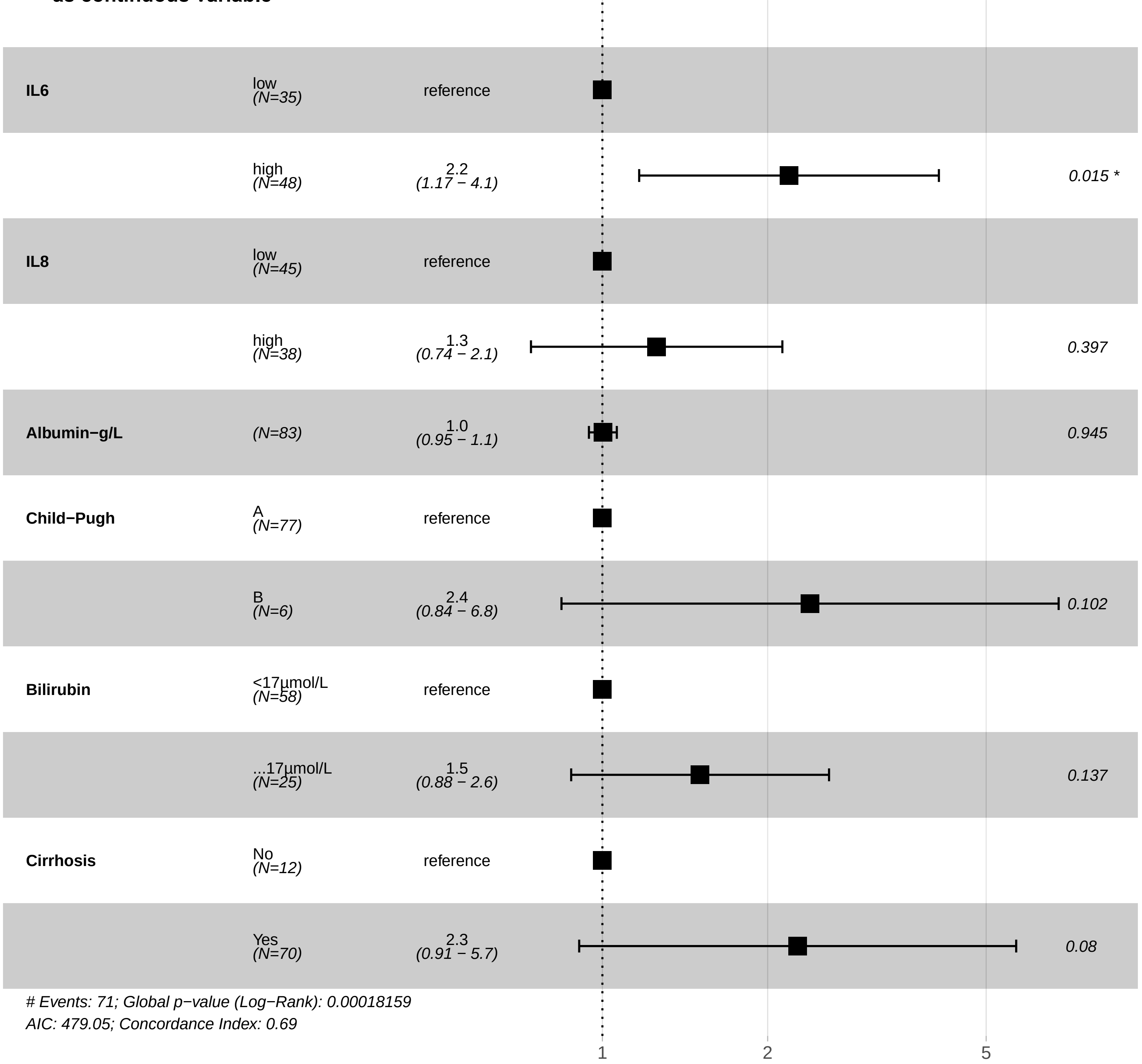

**Supplementary figure 2d.
Multivariable analysis using bilirubin
as continuous variable**

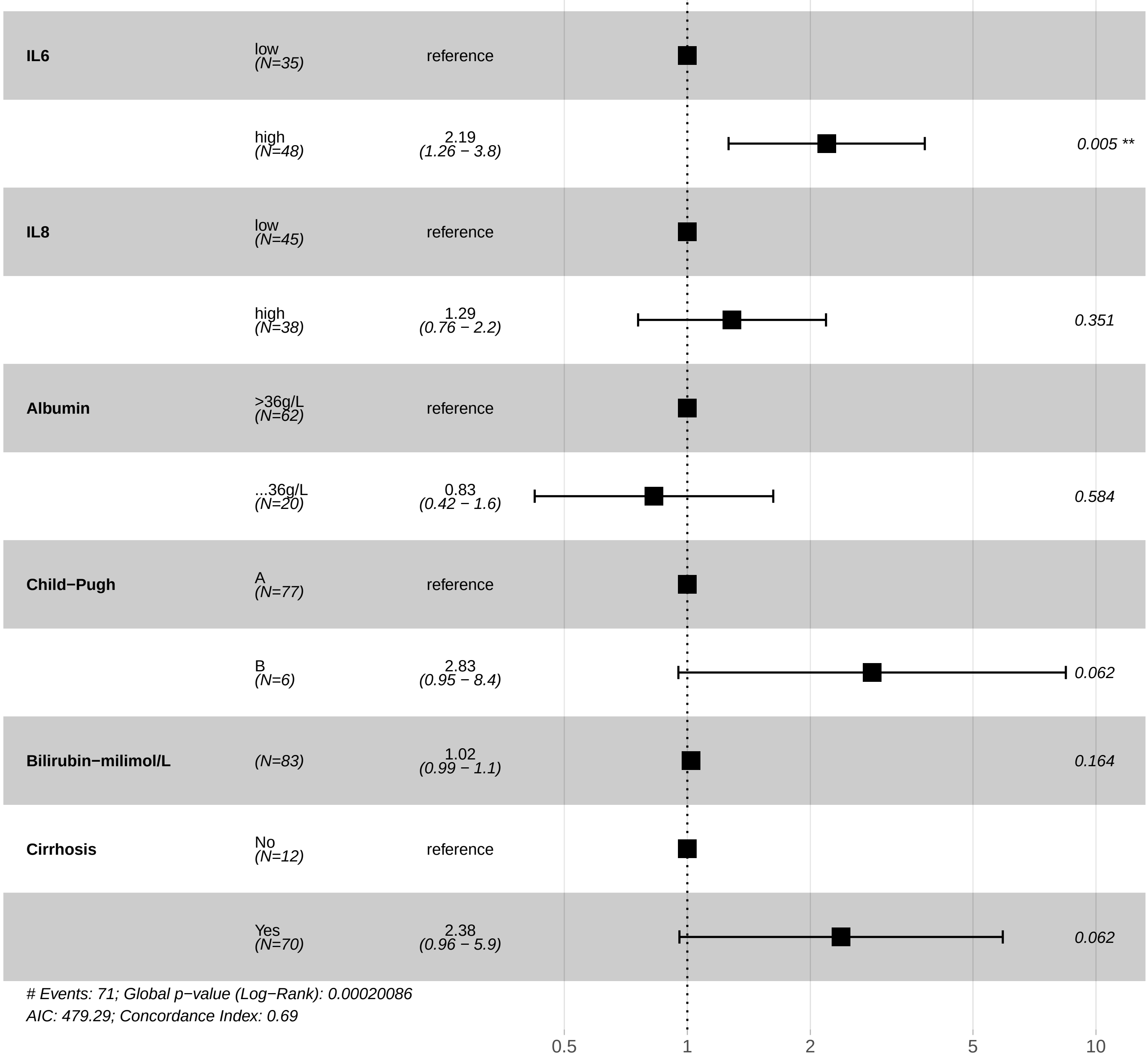

Supplementary figure 2e. Multivariable analysis using ALBI score as continuous variable

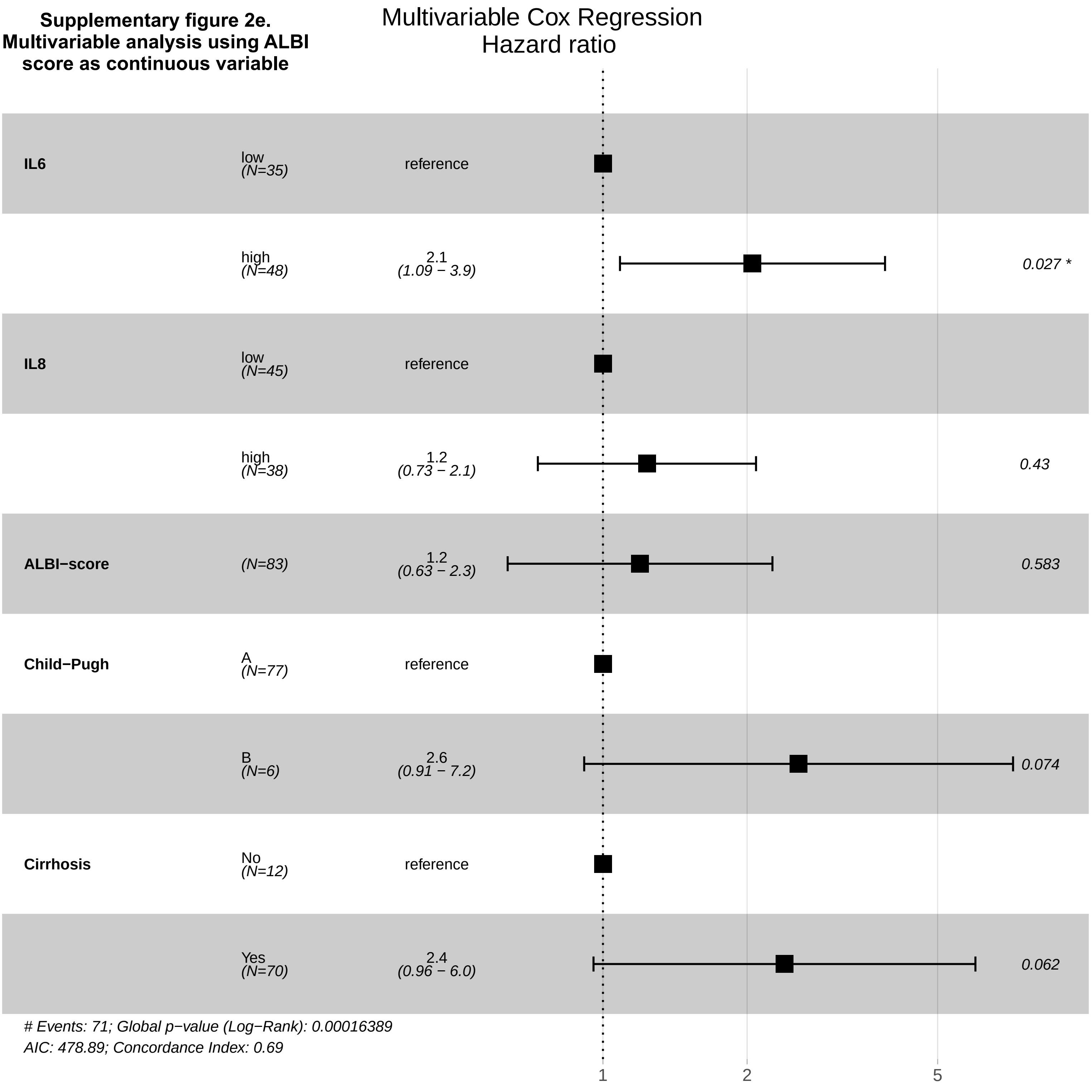

Supplement: Supplementary file 2 — Additional file 2. Supplementary figure 2. Results of seperate multivariable analysis models using following parameters as continous variables: (a) IL6, (b) IL8, (c) Albumin, (d) Bilirubin, (e) ALBI score. [file 13550_2021_791_MOESM2_ESM.pdf]
