# Supplementary material for: Prognostic value of baseline interleukin 6 levels in liver decompensation and survival in HCC patients undergoing radioembolization
Source: EJNMMI Res. 2021 Jun 2;11:51. doi: 10.1186/s13550-021-00791-w (PMC8172845; doi:10.1186/s13550-021-00791-w)
Supplement: Supplementary file 3 — Additional file 3. Supplementary figure 3. Kaplan-Meier curves showing cumulative probability of liver dysfunction without disease progression according to baseline IL6 (a) and IL8 (b) values. P values were calculated using the log rank test. [file 13550_2021_791_MOESM3_ESM.pdf]

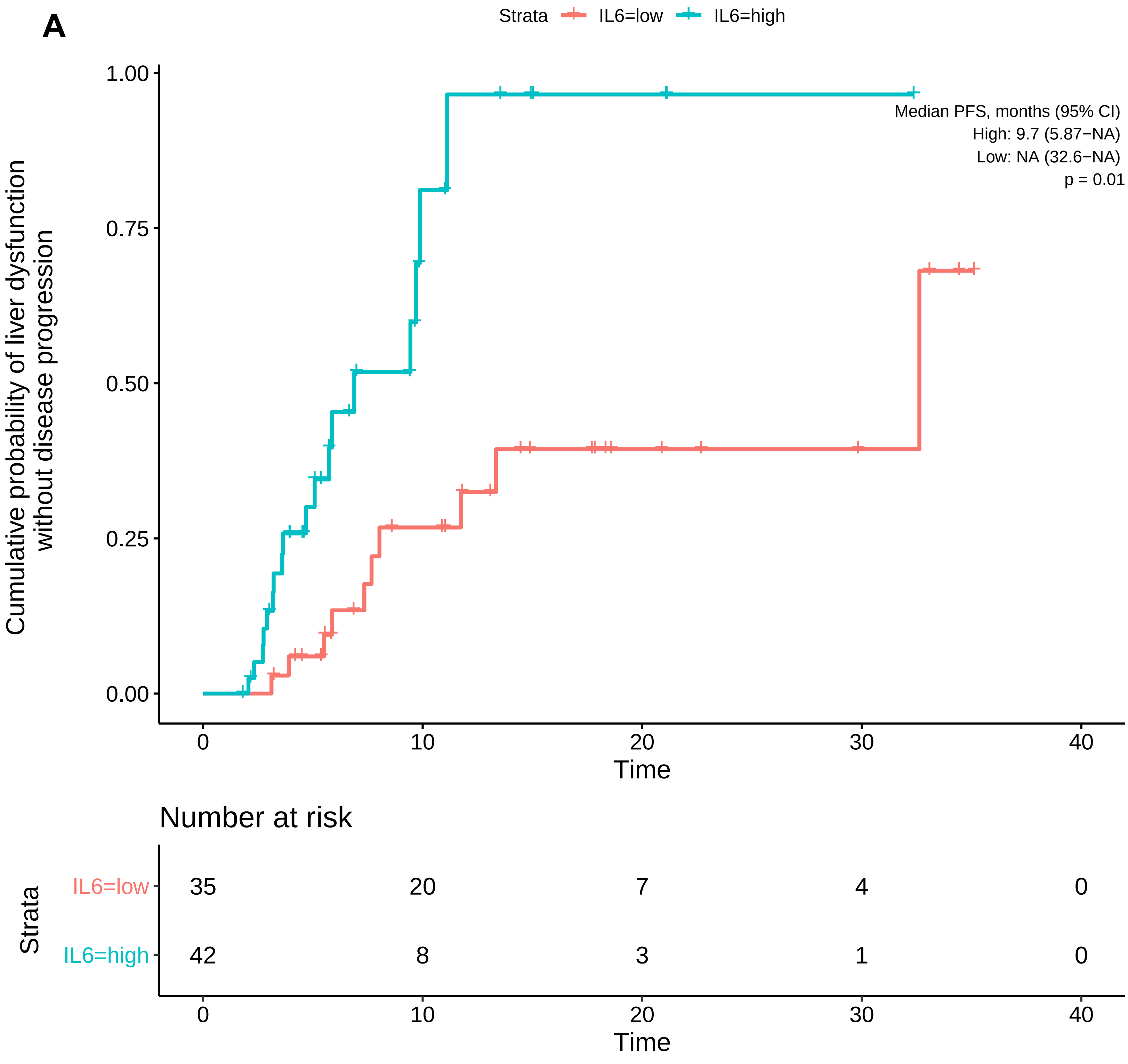
A
Strata
IL6=low
IL6=high
Cumulative probability of liver dysfunction without disease progression
1.00
0.75
0.50
0.25
0.00
Median PFS, months (95% CI)
High: 9.7 (5.87−NA)
Low: NA (32.6−NA)
p = 0.01
0
10
20
30
40
Time
Number at risk
Strata
IL6=low 35 20 7 4 0
IL6=high 42 8 3 1 0
0
10
20
30
40
Time

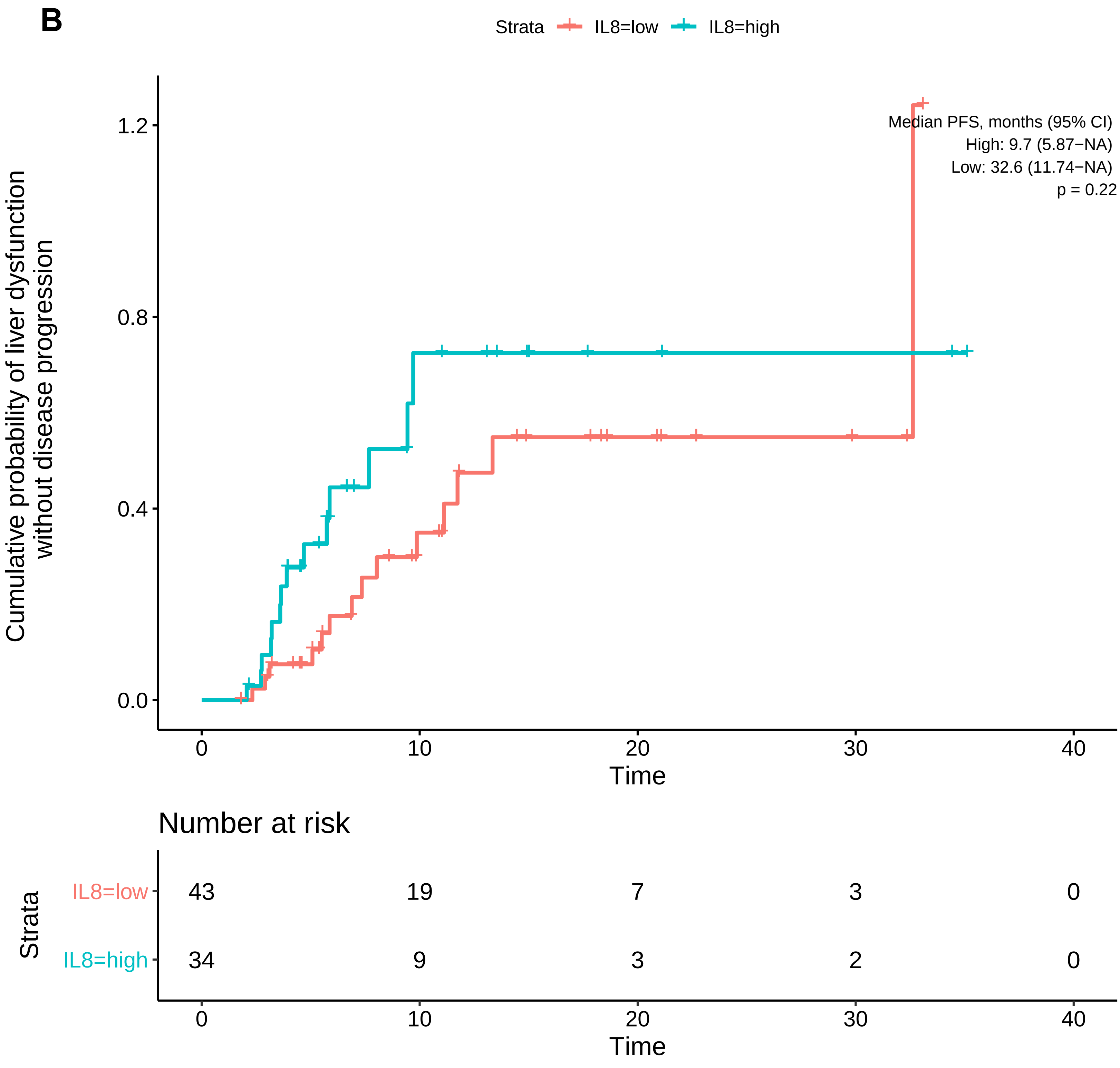
B
Strata
IL8=low
IL8=high
Median PFS, months (95% CI)
High: 9.7 (5.87−NA)
Low: 32.6 (11.74−NA)
p = 0.22
Cumulative probability of liver dysfunction
without disease progression
1.2
0.8
0.4
0.0
0
10
20
30
40
Time
Number at risk
Strata
IL8=low 43 19 7 3 0
IL8=high 34 9 3 2 0
0
10
20
30
40
Time
